# Role of maternal thyroid hormones in the developing neocortex and during human evolution

**DOI:** 10.3389/fnana.2013.00019

**Published:** 2013-07-16

**Authors:** Denise Stenzel, Wieland B. Huttner

**Affiliations:** Max Planck Institute of Molecular Biology and GeneticsDresden, Germany

**Keywords:** human brain evolution, developmental neurogenesis, cretinism, thyroid hormones, maternal hypothyroxinemia

## Abstract

The importance of thyroid hormones during brain development has been appreciated for many decades. In humans, low levels of circulating maternal thyroid hormones, e.g., caused by maternal hypothyroidism or lack of iodine in diet, results in a wide spectrum of severe neurological defects, including neurological cretinism characterized by profound neurologic impairment and mental retardation, underlining the importance of the maternal thyroid hormone contribution. In fact, iodine intake, which is essential for thyroid hormone production in the thyroid gland, has been related to the expansion of the brain, associated with the increased cognitive capacities during human evolution. Because thyroid hormones regulate transcriptional activity of target genes via their nuclear thyroid hormone receptors (THRs), even mild and transient changes in maternal thyroid hormone levels can directly affect and alter the gene expression profile, and thus disturb fetal brain development. Here we summarize how thyroid hormones may have influenced human brain evolution through the adaptation to new habitats, concomitant with changes in diet and, therefore, iodine intake. Further, we review the current picture we gained from experimental studies in rodents on the function of maternal thyroid hormones during developmental neurogenesis. We aim to evaluate the effects of maternal thyroid hormone deficiency as well as lack of THRs and transporters on brain development and function, shedding light on the cellular behavior conducted by thyroid hormones.

## INTRODUCTION

Thyroid hormones, that is, thyroxine (T4) and triiodothyronine (T3), are tyrosine-based hormones. All mammalian organisms maintain the same thyroid hormone structure, and in all vertebrates thyroid hormones are generated and stored in the same organ, the thyroid gland. Their synthesis requires iodine, and they are produced by follicle cells in the thyroid gland and secreted into the blood stream where they are transported to all cells in the organism (for details see Miot et al., 2012). Even though invertebrates do not have a thyroid gland, many organisms (including insects, plants, and algae) take up iodine from its environment and store iodine as a thyroid hormone precursor, that is, monoiodotyrosine (MIT) and diiodotyrosine (DIT) which result from iodinization of tyrosines (Dumont et al., 2011).

Upon binding to the thyroid hormone receptor (THR), the active thyroid hormone T3 act as transcriptional regulators by controlling gene expression. The predominant and best-characterized signaling pathway of T3 acts via their nuclear receptor, however, more recent studies describe non-genomic action of thyroid hormones via integrin a_v_b_3_, a cell membrane receptor (Bergh et al., 2005; Davis et al., 2008, 2011). The major functions of thyroid hormones in the adult is to regulate and increase the basic metabolic rate, and are involved in many biochemical reactions such as protein synthesis and enzymatic activity. Interestingly, however, thyroid hormones are fundamental for brain development and, more precisely, the maternal contribution of thyroxine is essential for the embryo to develop normal neurologic function and behavior. Given the ubiquitous (evolutionary conserved) presence of thyroid hormones in mammals and the deleterious defects in the absence of maternal thyroid hormones, we aim to (1) review the current concept on the role of thyroid hormones during brain evolution and (2) summarize the experimental models that helped to shed light on the underlying biological function of thyroid hormones during neurogenesis.

## POTENTIAL ROLE OF IODINE AND THYROID HORMONE IN HOMINID EVOLUTION

About 13 billion years ago, star explosion expelled all of the star’s material that was dispersed as “nuclear ash,” forming the foundation of our planet Earth and the basis of all elements. Iodine is a component originating from this supernova, and derived from a process of nucleosynthesis. Iodine is scarce in the earth’s surface, been washed away from terrestrial crust to the sea, which is enriched in iodine (Venturi et al., 2000; Venturi, 2011).

Interestingly, iodine is hypothesized to represent the most ancient source of anti-oxidant, and was present in primitive oxygenic cyanobacteria about 3.5 billion years ago. Recent studies found iodinated tyrosines (MIT and DIT) present in unicellular planktonic algae and in echinoid larvae. Sponges and corals contain large quantities of iodine and iodotyrosines (Dumont et al., 2011). The presence of thyroxine in fibrous exoskeletal scleroproteins of the lowest marine invertebrates (Venturi, 2011) may suggest that the original source of iodine and thyroid hormones in many animals might have been plants/algae, representing the main resource of food of marine organism, rather then endogenous production of thyroid hormones (Heyland and Moroz, 2005; Dumont et al., 2011), though endogenous synthesis of iodothyronines has been described for chordates (Dumont et al., 2011). The distribution of iodine varies geographically, with ocean and seawater presenting the major reservoir of iodine, while iodine concentrations decrease further inland and in mountainous regions. With the transfer of living organisms from the sea to land, evolutionary adaptation to iodine deficiency occurred (Venturi, 2011). The formation of the thyroid gland, allowing the synthesis of thyroid hormones from iodine and their storage, enabled the adaptation to the changing iodine concentration (from iodine-rich ocean to iodine-deficient terrestrial environment). Amphibian metamorphosis is completely dependent on thyroid hormones. Iodine, thyroid hormones, and their receptors are the essential metamorphic factors, transforming the aquatic vegetarian tadpole into a more complex terrestrial carnivore frog. Removal of the tadpole’s thyroid gland or inhibition of their thyroid hormone synthesis prevents metamorphosis (for review see Furlow and Neff, 2006).

Studies in the 1940s by Roth propose that iodine injection in the frog tadpole resulted in brain hypertrophy (Roth, 1946). Removal of the thyroid gland in frog tadpoles leads to brain atrophies, whereas engraftment of an additional thyroid gland increased brain expansion so much that the cranium sometimes bursts (Rey, 1948), reviewed in Venture and Bégin (2010) and Borensztejn (2005). However, these studies should be considered with caution as the experimental design and technical possibilities may have been extremely limited during that time and up to date, neither genetic nor pharmaceutical manipulation of thyroid hormones and their receptors could confirm the findings by Roth and Rey in the 1940s in tadpoles.

With the evolution of a primary brain in marine animals about 500–600 million years ago, thyroid cells originated from the primitive gut in vertebrates and specialized in the uptake and storage of iodo-compounds in a novel follicular thyroidal structure (reviewed by Crockford, 2009).

Deficiencies in thyroid hormones in pregnant women directly affect brain development of the fetus, resulting in neurological and neurocognitive disorders in human infants with defects ranging from decrease in intelligence and lethargy to mental retardation (Vermiglio et al., 1995; Morreale de Escobar et al., 2004; de Escobar et al., 2008; Berbel et al., 2009).

Normal human development strongly depends on (i) the intake of iodine, (ii) a functional thyroid gland, and (iii) maternal contribution of thyroxine during pregnancy. Inconstant and inadequate supply of iodine leads to goiter and cretinism. Goiter is the enlargement of the thyroid gland due to iodine deficiency in the diet. Cretinism is a medical condition due to dietary iodine deficiency, which results in severe pathologies including motor disabilities and neurological impairment. Congenital hypothyroidism is caused by the malfunction or absence of the thyroid gland, which produces thyroid hormones from iodine. More precisely, congenital hypothyroidism describes the deficiency in thyroid hormones as of prenatal onset of thyroid dysfunction and if diagnosed, can be treated by iodine supplementation and thyroxine (de Escobar et al., 2008; Berbel et al., 2009). Despite the introduction of iodized salt, to date several million people globally are affected by iodine deficiency, suffering from goiter and iodine deficiency-related brain damage and mental retardation, of which five million world-wide are diagnosed with cretinism (reviewed in Dobson, 1998).

Iodine intake due to diet dramatically impacted human evolution and possibly affected human brain expansion. Several factors may have had drastic impact on the iodine intake, such as (i) the colonization of hominid species further inland and in mountainous habitats, away from the iodine-rich oceans, (ii) the changing environmental condition such as temperature, as well as the (iii) dietary changes (from vegetarian to almost exclusively carnivorous diets).

Human brain evolution can be divided into four broad phases, that is, phase 1 Australopithecines, phase 2 *Homo (H.) habilis*, phase 3 *H. erectus* to early *H. sapiens*, and phase 4 early to present *H. sapiens *(Cro-Magnon type). Concomitant with the changes in habitat and consequently in diet during human evolution, relative brain size increased (that is the increase in brain weight relative to body weight) from Australopithecines to present *H. sapiens* (Cunnane and Crawford, 2003). While the average brain volume of Australopithecines was comparable to chimpanzee (450 g), the volume increased to around 650 g in *H. habilis*, 940 g in *H. erectus*, and 1400 g in *H. sapiens* (Cunnane and Crawford, 2003; Borensztejn, 2005).

Australopithecine habitat is thought to have changed from closed forest to open woodlands, with their predominant diet being mixed fruit, nuts, and smaller animals (e.g., small mammals, amphibians, and reptiles). The continuous consumption of high levels of thyroid hormones and iodine may have altered thyroid hormone rhythms, i.e., the concentration and/or periodic extent of maternal thyroid hormone contribution in the offspring, and induced changes in brain growth of the fetus. These modifications in embryo brain architecture may have been sufficient to stimulate brain expansion concomitant with a prolongation of fetal development, thereby extending the duration of neurogenesis and leading the way to the emergence of *H. habilis* (reviewed in Venture and Bégin, 2010; Venturi, 2011). *H. habilis* invaded more open habitats with abundant mammalian species, as dietary resources. Therefore, their diets were rich in brain-selective nutrients, including polyunsaturated fatty acids that are necessary for brain development and function (Cunnane and Crawford, 2003). This diet, enriched in fat and meat, further sustained the higher demand of energy of a bigger brain (Cunnane and Crawford, 2003). While their dietary effects on brain size expansion may have not been as drastic as observed for Australopithecines, the diet may still have been advantageous to support behavior traits. Although Australopithecines may have used wooden sticks, animal bones, and horns as fishing tools, * H. habilis* was recognized to be the first hominid to use stone to make tools, and the first human ancestor to show traces of asymmetry in the brain’s left hemisphere that is associated with development of areas for speech and language.

The evolution from *H. erectus* to present *H. sapiens* is further characterized by expansion of relative brain weight, concomitant with increased cognitive abilities and hunting skills. Further, the evolution of early *H. sapiens* coincided with major global climate change, plausibly resulting in crucial changes in many habitats and animal population, again resulting in alteration of *H. sapiens* diet depending on more intensified hunting and fishing. The increased consumption of dietary iodine and thyroid hormones may have triggered further shifts in thyroid hormone rhythms and promoted reproduction. Shore-based habitats further positively influenced iodine intake, and selection pressure would have favored individuals with thyroid hormone rhythms adapted to function best in new environmental conditions and habitats (for details see Crockford, 2003; Venture and Bégin, 2010).

Interestingly, Dobson (1998) proposed that Neanderthals suffered iodine deficiency disorders, possibly caused by the inland environment or by genetic differences of their thyroid gland compared to modern *H. sapiens*. The cold climate might have forced Neanderthals to move further inland, away from thyroid hormone and iodine-rich shore lands and diet, resulting in cretinism due to iodine deficiency. Distinctive Neanderthal skeletal traits are identical to those of modern humans that suffer from cretinism. And vice versa, human cretin skeletons resemble Neanderthals much more than modern humans (Dobson, 1998). Dobson postulates that a single genetic alteration, which improved the ability of the thyroid gland to uptake and utilize iodine, may account for differences between Neanderthals and modern humans.

Recently, studies by Obendorf and Oxnard hypothesize that *H. floresiensis*, a pygmy-sized, microcephalic hominin who lived from 95,000 to 13,000 years ago in Liang Bua on Flores (Indonesian Island), were myxoedematous endemic (ME) cretins, part of an inland population of (mostly unaffected) *H. sapiens*. ME cretins display symptoms of clinical hypothyroidism and are born without a functional thyroid due to iodine deficiency and subsequent lesion of thyroid. The lack of maternal and fetal thyroid hormones during the second half of gestation and in young infants up to 3 years of age results in dwarfism and reduced brain size. However, this condition is less severe than neurological cretinism which is characterized by severe neurological lesions without clinical hypothyroidism caused by severe iodine deficiency resulting in low maternal thyroid hormones during first half of gestation (Obendorf et al., 2008). Further examinations of cretin postcrania reveal evidence that cretins share numerous postcranial features with *H. floresiensis* (Oxnard et al., 2010, 2012). In agreement with these findings, analyses of human genetic variation revealed genomic regions that show signs of selection in the short-statured African Pygmy group; two of these regions concern the thyroid hormone pathway. Pygmies may have adapted to iodine-deficient areas, i.e., forest by genetic changes in their thyroid hormone pathway (Lopez Herraez et al., 2009). Although it is tempting to speculate that adaptation to natural iodine occurrence may have had an influence on brain evolution, clear molecular and genomic evidence is still lacking and needs further experimental proof.

The increasing complexity of the brain is further achieved by neuronal maturation and formation of connections. A major function of thyroid hormones is neuron and oligodendrocyte differentiation and maturation, axonal and dendrite growth, synaptogenesis and myelination. Recent studies reported a developmentally prolonged myelination in human neocortex compared to chimpanzee. Examination of length and density of myelinated axons and expression of myelin-related proteins revealed to be protracted in humans (Miller et al., 2012), which may be the result of time- and dose-dependent species-specific effects of thyroid hormones. The amount of thyroid hormone secretion and its timing, as well as the pulsatile rhythm of its production, is species-specific (reviewed and summarized in Crockford, 2003). High resolution proteomic analysis of adult human and chimpanzee blood plasma and cerebrospinal fluid samples revealed – despite very few species-specific differences, indicating a remarkable degree of conservation during 12 million years of evolution – a twofold increase in chimpanzee transthyretin, a thyroid hormone transporter expressed by the choroid plexus (Gagneux et al., 2001). Further, humans have lower plasma concentration of free, unbound T3 and T4, but higher total concentration of T4 than chimpanzee. Lower T4 to T3 ratio in chimpanzee, together with the skeletal traits and growth-retarded anatomical features, might reflect an iodine-deficient and hypothyroidism condition seen in modern humans (Previc, 2002). Tyrosine hydroxylase levels are positively regulated by thyroid hormones (Claustre et al., 1996), thus deficiency in thyroid hormones reduces the conversion of tyrosine to dopa, the precursor to dopamine. Dopaminergic activity is linked to various intellectual skills, and it is tempting to speculate that an evolutionary increase in thyroxine (T4) production and, therefore, increased dopaminergic action in humans compared to chimpanzee, may have lead the way to increased intellectual capability and higher cognitive abilities (Previc, 2002). Ideally, to explore human-specific differences in thyroid hormone expression and the transcriptional regulation of its target genes that may have played a role in brain evolution, one would like to analyze the transcriptomes of neurogenic developmental stages in chimpanzee and human fetal brain.

The turnover rates of thyroid hormones are distinct in many species. Interestingly, turnover times in Neanderthals are thought to be similar to modern dogs and cats, and therefore, more than ten-times faster compared to *H. sapiens* (Kaptein et al., 1994); (Venturi, 2011). These species-specific differences in thyroid hormone rhythm offer an evolutionary model of heterochronic specification during hominid evolution.

In human (and most vertebrate species), the growth, maturation, and morphological outcome of the offspring is directly influenced by thyroid hormones provided by the mother passing through the placenta, thereby controlling the growth and development of the fetus. Therefore, alteration in thyroid hormone concentration and availability (i.e., environmental changes and iodine accessibility) could translate into physiological and developmental responses in the offspring (thereby, connecting individuals without direct genetic modification) leading to effective genetic variations in the offspring that, in turn, mediates speciation events (i.e., expansion of brain size; Crockford, 2003, 2009; Venture and Bégin, 2010). Thyroid hormones, in particular T3 act via nuclear THRs to regulate gene expression, including nerve and epidermal growth factors that are crucial regulators of brain development (Giordano et al., 1992; Venture and Bégin, 2010). Additionally, many genes are involved in cell cycle exit and differentiation, and in controlling growth and development. Therefore, changing thyroid hormone levels can have the same effect as a mutation to these genes themselves, suggesting an evolutionary control mechanism in all vertebrates (Crockford, 2003).

## CELL BIOLOGICAL EFFECTS OF THYROID HORMONES DURING MAMMALIAN NEUROGENESIS

The key question that arises is how thyroid hormones influence brain development. In the following, we will summarize the knowledge we have gained employing rodents as an experimental model system to interfere with (a) maternal thyroid hormone contribution and (b) genetic manipulation of thyroid hormone components, such as receptors and transporters.

One important aspect to note is the timing and duration of the neurogenic period and the fetal thyroid gland formation and its maturation in various species (Howdeshell, 2002). Cortical neurogenesis, which is the generation of neurons from cortical progenitor cells, occurs in humans from week 5–20 of gestation, the earliest secretion of fetal thyroid hormones, however, starts during mid-gestation around week 18–22 of gestation (Obregon et al., 2007). In contrast, the cortical neurogenic phase in rat takes place from embryonic day (E)11 to E21, with E18 being the onset of fetal thyroid hormone secretion. The peak of neural maturation and myelination, axon and dendrite formation as well as synaptogenesis occurs in rodents within the first postnatal weeks, thus fully relying on thyroid hormones of the fetus/newborn. Interestingly, this corresponds to the third trimester of gestation in humans and thus, the human fetus is still exposed to maternal thyroid hormones during the majority of cortical neurogenesis, neuronal migration, and early phases of neuronal maturation (axonogenesis and dendrogenesis). Remarkably, the human fetal thyroid gland is fully functional at birth, while in rats it still continues to develop during the first postnatal weeks (reviewed in Ahmed et al., 2008). It is tempting to speculate that the constant and – relatively speaking – longer supply of maternal thyroid hormones during human fetal brain development compared to rat might be an underlying cause for the evolutionary expansion of the human brain and the increased cognitive abilities.

Therefore, the following part of this review will focus on the maternal contribution of thyroid hormones during neurogenesis in the embryonic neocortex [and we refer to excellent reviews summarizing the effects of thyroid hormones during postnatal neurogenesis (Anderson et al., 2003; Ahmed et al., 2008)].

The mammalian neocortex is a six-layered structure that contains (as neural cell types) neurons, astrocytes, and oligodendrocytes. All these cell types present in the adult cortex arise from neuroepithelial (NE) cells that, during development, line the ventricle. NE cells transform into radial glia cells (RGCs) that initially proliferate and divide symmetrically to expand the progenitor cell pool. Over the course of neurogenesis, RGCs start to divide asymmetrically to give rise to either directly a neuron or, more frequently, to a progenitor that is committed to the neuronal cell lineage. Referring to the location of their mitosis, the above-mentioned progenitors are collectively referred to as apical progenitors (APs; Fietz and Huttner, 2011; Lui et al., 2011). AP cell bodies reside in the ventricular zone and, therefore, have ideal access to the major source of thyroid hormones present in the cerebral spinal fluid (CSF) produced and secreted from the choroid plexus (Dratman et al., 1991). Further, APs maintain apical–basal polarity. Thus, they not only have access to the thyroid hormone-rich CSF in the ventricle via their apical side, but also possess a basal process that contacts the basal lamina at the meninges, an environment also likely to be enriched in maternal thyroid hormones that have crossed the blood–brain barrier. Besides APs, there exists a second principal class of cortical progenitors, referred to collectively as basal progenitors (BPs). In contrast to APs, BPs display no apico-basal polarity and, therefore, have no direct access to the CSF. Moreover, only a subtype of BPs called basal RGC, which is rare in mouse and rat, may contact the basal lamina and have access to thyroid hormones if present there (Lui et al., 2011). BPs derive from asymmetric division of APs, move their cell body basally into the subventricular zone and in mouse in ≈90% of cases generate two neurons, which will migrate to the cortical plate (Noctor et al., 2004; Attardo et al., 2008).

A recent study by Mohan et al. (2012) addressed the effect of maternal thyroid hormones on progenitor proliferation during cortical development. The rate of neural progenitor proliferation, and subsequent neuronal differentiation, was impaired in the developing embryonic cerebral cortex of hypothyroid rats. Deficiency in maternal thyroid hormones reduced both, the AP and the BP population, however, with a greater effect on BP proliferation, which likely was due to the initial reduction of BP-generating APs. As a consequence, all neurons, including early- as well as late-born neurons, were reduced, leading to a reduction in overall cortical thickness (Mohan et al., 2012). Interestingly, THRs and transporters are up-regulated in hypothyroid embryonic cortices, indicating the presence of a protective fetal mechanism to maternal thyroid hormone deficiency. In essence, Mohan et al. (2012) showed that maternal thyroid hormones influence cortical progenitor cell behavior, highlighting the importance of maternal contribution to fetal brain development.

From an evolutionary perspective, the increased gestational length and neurogenic period combined with the longer supply of maternal thyroid hormones in human may have led to sustained progenitor proliferation and ultimately, to the increased neuronal output.

It is therefore interesting to note that most genetic manipulation of THRs, deiodinases, and transporters in rodents did not result in the dramatic neurological defects observed in humans. Patients with an inactivating mutation in the human X-linked MCT8 (monocarboxylate transporter 8) gene, a thyroid hormone transporter, suffer from severe psychomotor retardation resembling hypothyroid patients, whereas Mct8 deficiency in mice failed to replicate the neurological symptoms, indicating that human fetal brain development is more sensitive to thyroid hormone fluctuations (reviewed in Horn and Heuer, 2010; Grijota-Martinez et al., 2011).

Thyroid hormone receptors and -transporters are expressed in the developing brain, and become increasingly expressed with the progression of neurogenesis.

The nuclear THRs are encoded by the *THRA* and *THRB* gene, which are transcribed into several isoforms. TH receptors alpha and beta display distinct spatiotemporal expression patterns in the developing mammalian cortex (Bradley et al., 1989, 1992). THR alpha 1 and -2 are widely expressed, with highest expression in the cortical plate, the site of cortical neuron differentiation. THR beta 1 transcript is restricted in distribution, with prominent expression in zones of neuroblast proliferation such as the germinal trigone and the cortical ventricular layer (Bradley et al., 1992). Nuclear TH receptors are present from the early onset of embryonic brain development in rodent as well as in human (Iskaros et al., 2000). Mouse transcriptome analyses of proliferative neural progenitors compared to fate-restricted, neurogenic progenitors indicated that concomitant with neuronal lineage commitment, the TH receptor beta and thyroid hormone transporter, Mct8, are more highly expressed (Arai et al., 2011). Strikingly, however, thryoid hormone receptor alpha (THRA) and thryoid hormone receptor beta (THRB)-deficient mice do not phenocopy the deleterious neurodevelopmental defects observed in hypothyroid rodents (Gothe et al., 1999), suggesting compensatory effects by other regulatory mechanisms. Further evidence was provided by introducing a point mutation in THRA which acts as a dominant-negative receptor that can bind DNA and recruits transcription co-repressors but fails to interact with T3, leading to a phenotype similar to congenital hypothyroidism (Flamant and Quignodon, 2010). It has been suggested, therefore, that the presence of unliganded THRs bound to DNA and thereby repressing gene transcription might be the underlying cause for the effects mediated by hypothyroidism (Chatonnet et al., 2011; Fauquier et al., 2011; Bernal and Morte, 2012). This is further supported by a recent study analyzing the regulation of positive and negative genes in the cerebral cortex and striatum of THR knockout mice and hypothyroid mice, identifying THR alpha 1 a the predominant regulator (Gil-Ibanez et al., 2013). By contrast, the plain absence of any DNA-bound nuclear THR does not lead to gene repression and neurodevelopmental defects.

While it was previously assumed that thyroid hormones diffuse passively into the cell, it has now become clear that their concentration is tightly regulated in the fetal brain by (i) the cell membrane transporters, Mct8 and Mct10 and organic anion transporter 2 and 3 (Oatp2 and Oatp3) that actively transport thyroid hormones across the cell membrane into the cell (reviewed in Horn and Heuer, 2010; Patel et al., 2011), and (ii) local conversion of thyroxine, T4, to the biologically active T3 by deiodinase type 2 (D2). High levels of T4 and T3 are detected in the embryonic brain before the secretion of fetal thyroid hormones from the fetal thyroid gland, indicating the functional relevance of maternal thyroid hormone. Further, almost all T3 in the brain is produced by local conversion of maternal T4 by D2 (Grijota-Martinez et al., 2011). In fact, there is a preferential protection of the fetal brain from T3 deficiency that is mediated by maternal T4, but not maternal T3. Any condition lowering maternal T4 is harmful for the brain development of a hypothyroid fetus, and cannot be restored by T3 injection alone in the pregnant mother (Calvo et al., 1990). Expression studies revealed the presence of D2 in glial cells in the cerebral cortex of neonatal rats, with several fold up-regulation in hypothyroid rats compared to control (Guadano-Ferraz et al., 1999). However, D2 knockout mice display only mild neurodevelopmental phenotype, despite reduced T3 levels in the neonatal brain similar to hypothyroid rats, suggesting that important compensatory mechanisms must be in play in the brain to minimize functional abnormalities in the absence of D2 (Galton et al., 2007; Morte et al., 2010a).

The deiodinase type 3 (D3), which catalyzes the deiodination of T4 to reverse T3, the biologically inactive thyroid hormone T3, acts as a thyroid hormone inactivator. In contrast to D2, which removes one iodine atom from the outer ring structure of T4 to produce the biologically active T3, D3 removes one iodine atom from the inner ring structure. Further D3 catalyzes the deiodination of the active T3 to the inactive diiodothyronine (T2). D3 is highly expressed in the placenta at the maternal–fetal interface, suggesting its regulatory function in mediating and modifying the maternal input of thyroid hormones and by this, the thyroid hormone status, i.e., maternal T4 and T3 concentration in the fetus (Huang et al., 2003). Lack of D3 function in mouse leads to neonatal thyrotoxicosis and delayed clearance of T3, indicating a crucial role for D3 in maturation and function of the thyroid hormone regulatory mechanism (Hernandez et al., 2006, 2012).

Congenital hypothyroidism (i.e., failure to develop a functional thyroid gland in the fetus and, therefore, lack of thyroid hormone production) does not cause gross developmental abnormalities, if detected and subsequently treated with thyroxine supplementation after birth. In fact, introduction of screening for hypothyroidism and immediate supplementation with thyroid hormones result in normal physical and intellectual development (Ares et al., 2005; Moleti et al., 2008, 2009; Berbel et al., 2009). Therefore, presence of maternal thyroid hormones protects the fetus even in case of fetal thyroid dysfunction. The maternal contribution still presents an essential proportion of the thyroid hormone supply to the fetus, indicated by the circulating levels of maternal T4 in the fetus. This not only demonstrates the importance of maternal thyroid hormone during the entire period of fetal brain development, but also shows that the fetal brain can be protected from irreversible damage when fetal thyroid hormones are absent.

Mild and transient reduction of maternal thyroid hormones to 70% of normal thyroid hormone serum levels from E12 to E15 in rat results in abnormal neural migration and misplaced neurons present even in the young adult (Auso et al., 2004). Further, this study shows alteration in radial migration of glutamatergic (excitatory) neurons, but not GABAergic (inhibitory) neurons. Whereas in the rat cerebellum, hypothyroidism caused a decrease in the number of cerebellar GABAergic interneurons due to decreased proliferation and delayed differentiation of the precursor cells (Manzano et al., 2007), indicating different sensitivity of progenitors and neurons to thyroid hormones in the various parts of the brain. The relatively mild degree of maternal hypothyroxinemia already altered the organization of the neocortex, with around 20% of glutamatergic neurons in abnormal location, and resulted in functional neurologic deficit as evidenced by an increased frequency of abnormal responses to an acoustic stimulus (Auso et al., 2004). Phenotypically similar defects were observed in newborn pups of iodine-deficient rats, which were severely deficient in maternal T4 (Lavado-Autric et al., 2003). Iodine deficiency in the diet of pregnant rats as well as humans results in decreased circulating free T4, but no significant alteration in T3 levels (Escobar-Morreale et al., 1997; Lavado-Autric et al., 2003), indicating that maternal T4, but not maternal T3, is essentially required for normal fetal neural development (Calvo et al., 1990). This is in line with previous observations that maternal T4 is the major source of locally converted fetal T3 in the brain. BrdU labeling during mid-gestation (and before the onset of fetal thyroid hormone secretion at E18 in rat) revealed abnormally located neurons in the cortex and hippocampus, indicating alteration of neuronal migration during corticogenesis (Auso et al., 2004; Lavado-Autric et al., 2003). Mild to severe iodine deficiency in the diet of rats, resulting in maternal hypothyroxinemia, leads to cytoarchitectonic changes, such as blurred layering in the cortex, which might be the underlying cause of cerebral damage and impaired brain function, which has also been described in humans deficient of iodine (Lavado-Autric et al., 2003; Auso et al., 2004). Long-term observation of children born by mothers from a mild to moderate iodine-deficient geographic area has revealed impaired neuropsychological and cognitive performance, and 68% of children were diagnosed with attention deficit and hyperactivity disorder (Vermiglio et al., 2004). Despite normal thyroid hormone levels after birth, the children born in iodine-deficient areas developed neurological deficits, indicating that maternal thyroid hormone contribution during early pregnancy is essential and cannot be restored upon presence of fetal thyroid hormones. Even a delay of 6–10 weeks in iodine supplementation of hypothyroxinemic mothers at the beginning of gestation increased the risk of neurodevelopmental delay in the progeny (Berbel et al., 2009).

Manipulation of thyroid hormone levels in pregnant rats before the onset of fetal thyroid function has been shown to affect gene expression in the fetal rat brain, indicating that maternal thyroid hormones can directly influence the gene expression during fetal neurogenesis (Dowling et al., 2000; Morte et al., 2010b). The genes identified by differential display analysis were selectively expressed in brain regions known to contain THRs, which act as ligand-activated transcription factors. Severe maternal hypothyroidism alters the gene expression of cerebral genes during mid-gestation, and responds rapidly to a single injection of T4 into dams (Berbel et al., 2010; Dowling et al., 2000). Dose-dependent differences in gene expression profiles were also evident when comparing varying degrees of thyroid hormone deficiency (mild to severe hypothyroidism) in rat hippocampus and neocortex (Royland et al., 2008), indicating different responses to thyroid hormones in different brain areas and at different developmental stages. Interestingly, the growth arrest and DNA-damage-inducible 45 b (Gadd45b) gene is differentially regulated by thyroid hormones (Dong et al., 2005). Gadd45b has been shown to link neural circuit activity to epigenetic DNA modification and expression of secreted factors important during adult neurogenesis (Ma et al., 2009). Recently, a comparison of human-specific deletion in non-coding sequences compared to chimpanzee and other mammals identified a region near the Gadd45g that has been correlated with the expansion of the forebrain region in humans (McLean et al., 2011). It is interesting to note that thyroid hormones may have an effect on gene expression of evolutionary distinctly regulated genes involved in brain expansion, especially in the context that hominid ancestors are hypothesized to reflect modern hypothyroid humans, i.e., cretins.

Interestingly, a recent study by Chatonnet et al. (2013) found a receptor-selective regulation of T3 target genes in neural cells, suggesting that evolutionary divergence of THRs alpha and beta may have occurred. It would be interesting to further investigate the presence and responsiveness of THR alpha and beta and the regulation of their distinct target genes during human brain evolution.

Thyroid hormone receptor binding sites and target genes that have been identified govern many genes involved in neuron development and neuronal migration, e.g., Neurogenin 2, Reelin receptor Vldlr, as well as signal transduction (Dong et al., 2009). The function of Reelin in neuronal migration has been well-characterized, and recently Pathak et al. (2011) demonstrated transcriptional control of Reelin by thyroid hormones through the presence of an intronic thyroid hormone response element. Deficiency of maternal thyroid hormones resulted in down-regulation of Reelin and, consequently, a loss of neuronal bipolarity and impaired neuronal migration (Pathak et al., 2011). Because actin and microtubule polymerization is affected by thyroid hormones (Leonard and Farwell, 1997), the lack of F-actin assembly and, therefore, changes in cytoskeleton organization in hypothyroid rats (Silva et al., 2006) may be an additional cause for the defects in neural cell migration. Further, thyroid hormones modify the expression of many extracellular matrix (ECM) molecules present in the cerebral cortex during neurogenesis, i.e., laminin, fibronectin, tenascin-C as well as adhesion molecules, such as neural cell adhesion molecule (NCAM; Iglesias et al., 1996; Alvarez-Dolado et al., 1998; Farwell and Dubord-Tomasetti, 1999; Lin et al., 2004). Alteration of ECM composition may directly affect neuronal migration and lead to the ectopically located neurons in damns of iodine-deficient mothers. Abnormal neuronal migration and maturation can ultimately lead to aberrant circuits, which can underlie an impaired brain function and neurological disorders.

## CONCLUSION

In summary, in the last decades many studies addressed the function of maternal thyroid hormones and its effects on target gene expression and transcriptional control, and ultimately, the consequences on cell behavior. It has become clear that the maternal contribution is essential for the normal development of a fetal brain, and we begin to understand and unravel the complex roles of thyroid hormones in brain development, and its implication during brain evolution. First, it is an interesting hypothesis that the evolutionary adaptation to new habitats and with this, the adaptation to new diets and iodine intake might be an underlying cause for the evolutionary expansion of the brain. The initial transition of animals from sea to land was accompanied with the formation of the thyroid gland, representing a reservoir for the storage of iodine when adjusting to iodine-deficient habitats. Second, iodine and thyroid hormones are indispensable for frog metamorphosis, and their deficiency has deleterious effects on fetal brain development in mammalian species. Third, hominid evolution, which is characterized by increased cognitive skills and abilities due to the drastically enlarged neocortex, has been linked to iodine availability, which is further underlined by the phenotypical similarities of Neanderthals and the pygmy-sized, microcephalic *H. floresiensis* with modern human cretins. Crockford (2003) hypothesized and formulated the thyroid rhythm theory, which predicts that when an ancestral species colonizes a radically new habitat with novel stress-producing characteristics, descendant species will be morphologically distinct but genetically similar until mutations have had a chance to accumulate. Thereby, it is not so much the genetic regulation and alteration in gene expression by thyroid hormones that resulted in the evolutionary expansion of the brain, but much more the tweaking of thyroid hormones in a dose- and time-dependent fashion and thereby exhibiting different effects on different brain regions that enable the increase in cognitive abilities.

## Conflict of Interest Statement

The authors declare that the research was conducted in the absence of any commercial or financial relationships that could be construed as a potential conflict of interest.

## References

[B1] Ahmed O. M. El-Gareib A. W. El-Bakry A. M. Abd El-Tawab S. M. Ahmed R. G. (2008). Thyroid hormones states and brain development interactions. *Int. J. Dev. Neurosci.* 26 147–209 10.1016/j.ijdevneu.2007.09.011 18031969

[B2] Alvarez-Dolado M. Gonzalez-Sancho J. M. Bernal J. Munoz A. (1998). Developmental expression of the tenascin-C is altered by hypothyroidism in the rat brain. *Neuroscience* 84 309–322 10.1016/S0306-4522(97)00511-3 9580330

[B3] Anderson G. W. Schoonover C. M. Jones S. A. (2003). Control of thyroid hormone action in the developing rat brain. *Thyroid* 13 1039–1056 10.1089/105072503770867219 14651788

[B4] Arai Y. Pulvers J. N. Haffner C. Schilling B. Nusslein I. Calegari F. (2011). Neural stem and progenitor cells shorten S-phase on commitment to neuron production. *Nat. Commun.* 2 154 10.1038/ncomms1155 PMC3105305 21224845

[B5] Ares S. Quero J Morreale de Escobar G. (2005). Neonatal iodine deficiency: clinical aspects. *J. Pediatr. Endocrinol. Metab.* 18(Suppl. 1) 1257–1264 10.1515/JPEM.2005.18.S1.1257 16398457

[B6] Attardo A. Calegari F. Haubensak W. Wilsch-Brauninger M. Huttner W. B. (2008). Live imaging at the onset of cortical neurogenesis reveals differential appearance of the neuronal phenotype in apical versus basal progenitor progeny. *PLoS ONE * 3:e2388 10.1371/journal.pone.0002388 PMC2398773 18545663

[B7] Auso E. Lavado-Autric R. Cuevas E. Del Rey F. E. Morreale De Escobar G. Berbel P. (2004). A moderate and transient deficiency of maternal thyroid function at the beginning of fetal neocorticogenesis alters neuronal migration. *Endocrinology* 145 4037–4047 10.1210/en.2004-0274 15087434

[B8] Berbel P. Mestre J. L. Santamaria A. Palazon I. Franco A. Graells M. (2009). Delayed neurobehavioral development in children born to pregnant women with mild hypothyroxinemia during the first month of gestation: the importance of early iodine supplementation. *Thyroid* 19 511–519 10.1089/thy.2008.0341 19348584

[B9] Berbel P. Navarro D. Auso E. Varea E. Rodriguez A. E. Ballesta J. J. (2010). Role of late maternal thyroid hormones in cerebral cortex development: an experimental model for human prematurity. *Cereb. Cortex* 20 1462–1475 10.1093/cercor/bhp212 19812240 PMC2871377

[B10] Bergh J. J. Lin H. Y. Lansing L. Mohamed S. N. Davis F. B. Mousa S. (2005). Integrin alphaVbeta3 contains a cell surface receptor site for thyroid hormone that is linked to activation of mitogen-activated protein kinase and induction of angiogenesis. *Endocrinology* 146 2864–2871 10.1210/en.2005-0102 15802494

[B11] Bernal J. Morte B. (2012). Thyroid hormone receptor activity in the absence of ligand: physiological and developmental implications. *Biochim. Biophys. Acta* 1830 3893–3899 10.1016/j.bbagen.2012.04.014 22554916

[B12] Borensztejn S. (2005). The Iodine Probabilistic Influence in the Biological Evolution.

[B13] Bradley D. J. Towle H. C Young W. S. III (1992). Spatial and temporal expression of alpha- and beta-thyroid hormone receptor mRNAs, including the beta 2-subtype, in the developing mammalian nervous system. *J. Neurosci.* 12 2288–2302 1607941 10.1523/JNEUROSCI.12-06-02288.1992 PMC6575910

[B14] Bradley D. J. Young W. S. III Weinberger C. (1989). Differential expression of alpha and beta thyroid hormone receptor genes in rat brain and pituitary. *Proc. Natl. Acad. Sci. U.S.A.* 86 7250–7254 10.1073/pnas.86.18.7250 2780568 PMC298035

[B15] Calvo R. Obregon M. J. Ruiz de Ona C. Escobar del Rey F Morreale de Escobar G. (1990). Congenital hypothyroidism, as studied in rats. Crucial role of maternal thyroxine but not of 3,5,3′-triiodothyronine in the protection of the fetal brain. *J. Clin. Invest.* 86 889–899 10.1172/JCI114790 2394838 PMC296808

[B16] Chatonnet F. Guyot R. Benoit G. Flamant F. (2013). Genome-wide analysis of thyroid hormone receptors shared and specific functions in neural cells. *Proc. Natl. Acad. Sci. U.S.A.* 110 E766–E775 10.1073/pnas.1210626110 23382204 PMC3581916

[B17] Chatonnet F. Picou F. Fauquier T. Flamant F. (2011). Thyroid hormone action in cerebellum and cerebral cortex development. *J. Thyroid Res. * 2011:145762 10.4061/2011/145762 PMC3134109 21765985

[B18] Claustre J. Balende C. Pujol J. F. (1996). Influence of the thyroid hormone status on tyrosine hydroxylase in central and peripheral catecholaminergic structures. *Neurochem. Int.* 28 277–281 10.1016/0197-0186(95)00088-7 8813245

[B19] Crockford S. J. (2003). Thyroid rhythm phenotypes and hominid evolution: a new paradigm implicates pulsatile hormone secretion in speciation and adaptation changes. *Comp. Biochem. Physiol. A Mol. Integr. Physiol.* 135 105–129 10.1016/S1095-6433(02)00259-3 12727549

[B20] Crockford S. J. (2009). Evolutionary roots of iodine and thyroid hormones in cell–cell signaling. *Integr. Comp. Biol.* 49 155–166 10.1093/icb/icp053 21669854

[B21] Cunnane S. C. Crawford M. A. (2003). Survival of the fattest: fat babies were the key to evolution of the large human brain. *Comp. Biochem. Physiol. A Mol. Integr. Physiol.* 136 17–26 10.1016/S1095-6433(03)00048-5 14527626

[B22] Davis P. J. Davis F. B. Mousa S. A. Luidens M. K. Lin H. Y. (2011). Membrane receptor for thyroid hormone: physiologic and pharmacologic implications. *Annu. Rev. Pharmacol. Toxicol.* 51 99–115 10.1146/annurev-pharmtox-010510-100512 20868274

[B23] Davis P. J. Leonard J. L. Davis F. B. (2008). Mechanisms of nongenomic actions of thyroid hormone. *Front. Neuroendocrinol.* 29 211–218 10.1016/j.yfrne.2007.09.003 17983645

[B24] de Escobar G. M. Ares S. Berbel P. Obregon M. J del Rey F. E. (2008). The changing role of maternal thyroid hormone in fetal brain development. *Semin. Perinatol.* 32 380–386 10.1053/j.semperi.2008.09.002 19007674

[B25] Dobson J. E. (1998). The iodine factor in health and evolution. *Geogr. Rev.* 88 1–28 10.2307/215869

[B26] Dong H. Wade M. Williams A. Lee A. Douglas G. R. Yauk C. (2005). Molecular insight into the effects of hypothyroidism on the developing cerebellum. *Biochem. Biophys. Res. Commun.* 330 1182–1193 10.1016/j.bbrc.2005.03.099 15823568

[B27] Dong H. Yauk C. L. Rowan-Carroll A. You S. H. Zoeller R. T. Lambert I. (2009). Identification of thyroid hormone receptor binding sites and target genes using ChIP-on-chip in developing mouse cerebellum. *PLoS ONE * 4:e4610 10.1371/journal.pone.0004610 PMC2643481 19240802

[B28] Dowling A. L. Martz G. U. Leonard J. L. Zoeller R. T. (2000). Acute changes in maternal thyroid hormone induce rapid and transient changes in gene expression in fetal rat brain. *J. Neurosci.* 20 2255–2265 10704501 10.1523/JNEUROSCI.20-06-02255.2000 PMC6772490

[B29] Dratman M. B. Crutchfield F. L. Schoenhoff M. B. (1991). Transport of iodothyronines from bloodstream to brain: contributions by blood:brain and choroid plexus:cerebrospinal fluid barriers. *Brain Res.* 554 229–236 10.1016/0006-8993(91)90194-Z 1933305

[B30] Dumont J. Opitz R. Christophe D. Vassart G. Roger P. P Maenhaut C. (2011). *Ontogeny, Anatomy, Metabolism and Physiology of the Thyroid* ed. Groot L. J. D. Available at

[B31] Escobar-Morreale H. F. Obregon M. J. Hernandez A. Escobar del Rey F Morreale de Escobar G. (1997). Regulation of iodothyronine deiodinase activity as studied in thyroidectomized rats infused with thyroxine or triiodothyronine. *Endocrinology* 138 2559–2568 10.1210/en.138.6.2559 9165049

[B32] Farwell A. P. Dubord-Tomasetti S. A. (1999). Thyroid hormone regulates the extracellular organization of laminin on astrocytes. *Endocrinology* 140 5014–5021 10.1210/en.140.11.5014 10537126

[B33] Fauquier T. Romero E. Picou F. Chatonnet F. Nguyen X. N. Quignodon L. (2011). Severe impairment of cerebellum development in mice expressing a dominant-negative mutation inactivating thyroid hormone receptor alpha1 isoform. *Dev. Biol.* 356 350–358 10.1016/j.ydbio.2011.05.657 21621530

[B34] Fietz S. A. Huttner W. B. (2011). Cortical progenitor expansion, self-renewal and neurogenesis-a polarized perspective. *Curr. Opin. Neurobiol.* 21 23–35 10.1016/j.conb.2010.10.002 21036598

[B35] Flamant F. Quignodon L. (2010). Use of a new model of transgenic mice to clarify the respective functions of thyroid hormone receptors in vivo. *Heart Fail. Rev.* 15 117–120 10.1007/s10741-008-9121-y 19137427

[B36] Furlow J. D. Neff E. S. (2006). A developmental switch induced by thyroid hormone: *Xenopus laevis* metamorphosis. *Trends Endocrinol. Metab.* 17 40–47 10.1016/j.tem.2006.01.007 16464605

[B37] Gagneux P. Amess B. Diaz S. Moore S. Patel T. Dillmann W. (2001). Proteomic comparison of human and great ape blood plasma reveals conserved glycosylation and differences in thyroid hormone metabolism. *Am. J. Phys. Anthropol.* 115 99–109 10.1002/ajpa.1061 11385598

[B38] Galton V. A. Wood E. T. St Germain E. A. Withrow C. A. Aldrich G. St Germain G. M. (2007). Thyroid hormone homeostasis and action in the type 2 deiodinase-deficient rodent brain during development. *Endocrinology* 148 3080–3088 10.1210/en.2006-1727 17332058

[B39] Gil-Ibanez P. Morte B. Bernal J. (2013). Role of thyroid hormone receptor subtypes alpha and beta on gene expression in the cerebral cortex and striatum of postnatal mice. *Endocrinology* 154 1940–1947 10.1210/en.2012-2189 23493375

[B40] Giordano T. Pan J. B. Casuto D. Watanabe S. Arneric S. P. (1992). Thyroid hormone regulation of NGF, NT-3 and BDNF RNA in the adult rat brain. *Brain Res. Mol. Brain Res.* 16 239–245 10.1016/0169-328X(92)90231-Y 1337933

[B41] Gothe S. Wang Z. Ng L. Kindblom J. M. Barros A. C. Ohlsson C. (1999). Mice devoid of all known thyroid hormone receptors are viable but exhibit disorders of the pituitary-thyroid axis, growth, and bone maturation. *Genes Dev.* 13 1329–1341 10.1101/gad.13.10.1329 10346821 PMC316730

[B42] Grijota-Martinez C. Diez D. Morreale de Escobar G. Bernal J. Morte B. (2011). Lack of action of exogenously administered T3 on the fetal rat brain despite expression of the monocarboxylate transporter 8. *Endocrinology* 152 1713–1721 10.1210/en.2010-1014 21303950

[B43] Guadano-Ferraz A. Escamez M. J. Rausell E. Bernal J. (1999). Expression of type 2 iodothyronine deiodinase in hypothyroid rat brain indicates an important role of thyroid hormone in the development of specific primary sensory systems. *J. Neurosci.* 19 3430–3439 10212303 10.1523/JNEUROSCI.19-09-03430.1999 PMC6782260

[B44] Hernandez A. Martinez M. E. Fiering S. Galton V. A St Germain D. (2006). Type 3 deiodinase is critical for the maturation and function of the thyroid axis. *J. Clin. Invest.* 116 476–484 10.1172/JCI26240 16410833 PMC1326144

[B45] Hernandez A. Morte B. Belinchon M. M. Ceballos A. Bernal J. (2012). Critical role of types 2 and 3 deiodinases in the negative regulation of gene expression by T(3)in the mouse cerebral cortex. *Endocrinology* 153 2919–2928 10.1210/en.2011-1905 22523155 PMC3359606

[B46] Heyland A. Moroz L. L. (2005). Cross-kingdom hormonal signaling: an insight from thyroid hormone functions in marine larvae. *J. Exp. Biol.* 208 4355–4361 10.1242/jeb.01877 16339856

[B47] Horn S. Heuer H. (2010). Thyroid hormone action during brain development: more questions than answers. *Mol. Cell. Endocrinol.* 315 19–26 10.1016/j.mce.2009.09.008 19765631

[B48] Howdeshell K. L. (2002). A model of the development of the brain as a construct of the thyroid system. *Environ. Health Perspect. * 110(Suppl. 3) 337–348 10.1289/ehp.02110s3337 12060827 PMC1241181

[B49] Huang S. A. Dorfman D. M. Genest D. R. Salvatore D. Larsen P. R. (2003). Type 3 iodothyronine deiodinase is highly expressed in the human uteroplacental unit and in fetal epithelium. *J. Clin. Endocrinol. Metab.* 88 1384–1388 10.1210/jc.2002-021291 12629133

[B50] Iglesias T. Caubin J. Stunnenberg H. G. Zaballos A. Bernal J. Munoz A. (1996). Thyroid hormone-dependent transcriptional repression of neural cell adhesion molecule during brain maturation. *EMBO J.* 15 4307–4316 8861959 PMC452156

[B51] Iskaros J. Pickard M. Evans I. Sinha A. Hardiman P. Ekins R. (2000). Thyroid hormone receptor gene expression in first trimester human fetal brain. *J. Clin. Endocrinol. Metab.* 85 2620–2623 10.1210/jc.85.7.2620 10902817

[B52] Kaptein E. M. Hays M. T. Ferguson D. C. (1994). Thyroid hormone metabolism. A comparative evaluation. *Vet. Clin. North Am. Small Anim. Pract.* 24 431–466 10.1172/JCI18236 8053105

[B53] Lavado-Autric R. Auso E. Garcia-Velasco J. V. Arufe Mdel C. Escobar del Rey F. Berbel P. (2003). Early maternal hypothyroxinemia alters histogenesis and cerebral cortex cytoarchitecture of the progeny. *J. Clin. Invest.* 111 1073–1082 10.1172/JCI200316262 12671057 PMC152582

[B54] Leonard J. L. Farwell A. P. (1997). Thyroid hormone-regulated actin polymerization in brain. *Thyroid* 7 147–151 10.1089/thy.1997.7.147 9086583

[B55] Lin K. H. Chen C. Y. Chen S. L. Yen C. C. Huang Y. H. Shih C. H. (2004). Regulation of fibronectin by thyroid hormone receptors. *J. Mol. Endocrinol.* 33 445–458 10.1677/jme.1.01505 15525600

[B56] Lopez Herraez D. Bauchet M. Tang K. Theunert C. Pugach I. Li J. (2009). Genetic variation and recent positive selection in worldwide human populations: evidence from nearly 1 million SNPs. *PLoS ONE * 4:e7888 10.1371/journal.pone.0007888 PMC2775638 19924308

[B57] Lui J. H. Hansen D. V. Kriegstein A. R. (2011). Development and evolution of the human neocortex. *Cell* 146 18–36 10.1016/j.cell.2011.06.030 21729779 PMC3610574

[B58] Ma D. K. Jang M. H. Guo J. U. Kitabatake Y. Chang M. L. Pow-Anpongkul N. (2009). Neuronal activity-induced Gadd45b promotes epigenetic DNA demethylation and adult neurogenesis. *Science* 323 1074–1077 10.1126/science.1166859 19119186 PMC2726986

[B59] Manzano J. Cuadrado M. Morte B. Bernal J. (2007). Influence of thyroid hormone and thyroid hormone receptors in the generation of cerebellar gamma-aminobutyric acid-ergic interneurons from precursor cells. *Endocrinology* 148 5746–5751 10.1210/en.2007-0567 17761765

[B60] McLean C. Y. Reno P. L. Pollen A. A. Bassan A. I. Capellini T. D. Guenther C. (2011). Human-specific loss of regulatory DNA and the evolution of human-specific traits. *Nature* 471 216–219 10.1038/nature09774 21390129 PMC3071156

[B61] Miller D. J. Duka T. Stimpson C. D. Schapiro S. J. Baze W. B. McArthur M. J. (2012). Prolonged myelination in human neocortical evolution. *Proc. Natl. Acad. Sci. U.S.A.* 109 16480–16485 10.1073/pnas.1117943109 23012402 PMC3478650

[B62] Miot F. Dupuy C. Dumont J. Rousset B. (2012). Thyroid hormone synthesis and secretion (Leslie J De Groot). Available at

[B63] Mohan V. Sinha R. A. Pathak A. Rastogi L. Kumar P. Pal A. (2012). Maternal thyroid hormone deficiency affects the fetal neocorticogenesis by reducing the proliferating pool, rate of neurogenesis and indirect neurogenesis. *Exp. Neurol.* 237 477–488 10.1016/j.expneurol.2012.07.019 22892247

[B64] Moleti M. Lo Presti V. P. Campolo M. C. Mattina F. Galletti M. Mandolfino M. (2008). Iodine prophylaxis using iodized salt and risk of maternal thyroid failure in conditions of mild iodine deficiency. *J. Clin. Endocrinol. Metab.* 93 2616–2621 10.1210/jc.2008-0352 18413422

[B65] Moleti M. Lo Presti V. P. Mattina F. Mancuso A. De Vivo A. Giorgianni G. (2009). Gestational thyroid function abnormalities in conditions of mild iodine deficiency: early screening versus continuous monitoring of maternal thyroid status. *Eur. J. Endocrinol.* 160 611–617 10.1530/EJE-08-0709 19179457

[B66] Morreale de Escobar G. Obregon M. J Escobar del Rey F. (2004). Role of thyroid hormone during early brain development. *Eur. J. Endocrinol.* 151(Suppl. 3) U25–U37 10.1530/eje.0.151U025 15554884

[B67] Morte B. Ceballos A. Diez D. Grijota-Martinez C. Dumitrescu A. M. Di Cosmo C. (2010a). Thyroid hormone-regulated mouse cerebral cortex genes are differentially dependent on the source of the hormone: a study in monocarboxylate transporter-8- and deiodinase-2-deficient mice. *Endocrinology* 151 2381–2387 10.1210/en.2009-0944 20211971 PMC2869252

[B68] Morte B. Diez D. Auso E. Belinchon M. M. Gil-Ibanez P. Grijota-Martinez C. (2010b). Thyroid hormone regulation of gene expression in the developing rat fetal cerebral cortex: prominent role of the Ca2+/calmodulin-dependent protein kinase IV pathway. *Endocrinology* 151 810–820 10.1210/en.2009-0958 20056827

[B69] Noctor S. C. Martinez-Cerdeno V. Ivic L. Kriegstein A. R. (2004). Cortical neurons arise in symmetric and asymmetric division zones and migrate through specific phases. *Nat. Neurosci.* 7 136–144 10.1038/nn1172 14703572

[B70] Obendorf P. J. Oxnard C. E. Kefford B. J. (2008). Are the small human-like fossils found on Flores human endemic cretins? *Proc. Biol. Sci.* 275 1287–1296 10.1098/rspb.2007.1488 18319214 PMC2602669

[B71] Obregon M. J. Calvo R. M. Del Rey F. E de Escobar G. M. (2007). Ontogenesis of thyroid function and interactions with maternal function. *Endocr. Dev.* 10 86–98 10.1159/000106821 17684391

[B72] Oxnard C. Obendorf P. J. Kefford B. J. (2010). Post-cranial skeletons of hypothyroid cretins show a similar anatomical mosaic as *Homo floresiensis*. *PLoS ONE* 5:e13018 10.1371/journal.pone.0013018 PMC2946357 20885948

[B73] Oxnard C. Obendorf P. J. Kefford B. J. Dennison J. (2012). More on the Liang Bua finds and modern human cretins. *Homo* 63 407–412 10.1016/j.jchb.2012.09.005 23107933

[B74] Patel J. Landers K. Li H. Mortimer R. H. Richard K. (2011). Thyroid hormones and fetal neurological development. *J. Endocrinol.* 209 1–8 10.1530/JOE-10-0444 21212091

[B75] Pathak A. Sinha R. A. Mohan V. Mitra K. Godbole M. M. (2011). Maternal thyroid hormone before the onset of fetal thyroid function regulates reelin and downstream signaling cascade affecting neocortical neuronal migration. *Cereb. Cortex* 21 11–21 10.1093/cercor/bhq052 20368265

[B76] Previc F. H. (2002). Thyroid hormone production in chimpanzees and humans: implications for the origins of human intelligence. *Am. J. Phys. Anthropol.* 118 402–403 10.1002/ajpa.10095 12124921

[B77] Royland J. E. Parker J. S. Gilbert M. E. (2008). A genomic analysis of subclinical hypothyroidism in hippocampus and neocortex of the developing rat brain. *J. Neuroendocrinol. * 20 1319–1338 10.1111/j.1365-2826.2008.01793.x 19094080

[B78] Silva F. G. Giannocco G. Santos M. F. Nunes M. T. (2006). Thyroid hormone induction of actin polymerization in somatotrophs of hypothyroid rats: potential repercussions in growth hormone synthesis and secretion. *Endocrinology* 147 5777–5785 10.1210/en.2006-0110 16990344

[B79] Venture S Bégin M. E. (2010). “Throid hormone, iodine and human brain evolution,” in *Human Brain Evolution* eds Cunnane S. Stewart K. (Hoboken: John Wiley & Sons, Inc.).

[B80] Venturi S. (2011). Evolutionary significance of iodine. *Curr. Chem. Biol.* 5 155–162

[B81] Venturi S. Donati F. M. Venturi A. Venturi M. (2000). Environmental iodine deficiency: a challenge to the evolution of terrestrial life? *Thyroid* 10 727–729 10.1089/10507250050137851 11014322

[B82] Vermiglio F. Lo Presti V. P. Moleti M. Sidoti M. Tortorella G. Scaffidi G. (2004). Attention deficit and hyperactivity disorders in the offspring of mothers exposed to mild-moderate iodine deficiency: a possible novel iodine deficiency disorder in developed countries. *J. Clin. Endocrinol. Metab.* 89 6054–6060 10.1210/jc.2004-0571 15579758

[B83] Vermiglio F. Lo Presti V. P. Scaffidi Argentina G. Finocchiaro M. D. Gullo D. Squatrito S. (1995). Maternal hypothyroxinaemia during the first half of gestation in an iodine deficient area with endemic cretinism and related disorders. *Clin. Endocrinol. (Oxf.)* 42 409–415 10.1111/j.1365-2265.1995.tb02650.x 7750195

